# Carbonyl Cyanide m-Chlorophenylhydrazine (CCCP) Reverses Resistance to Colistin, but Not to Carbapenems and Tigecycline in Multidrug-Resistant *Enterobacteriaceae*

**DOI:** 10.3389/fmicb.2017.00228

**Published:** 2017-02-14

**Authors:** John Osei Sekyere, Daniel G. Amoako

**Affiliations:** ^1^Division of Microbiology, Department of Pharmaceutics, Faculty of Pharmaceutical Sciences, Kwame Nkrumah University of Science and TechnologyKumasi, Ghana; ^2^Discipline of Pharmaceutical Sciences, School of Health Sciences, University of KwaZulu-NatalDurban, South Africa; ^3^Biomedical Resource Unit, School of Laboratory Medicine and Medical Sciences, University of KwaZulu-NatalDurban, South Africa

**Keywords:** carbapenem, efflux pumps, colistin, CCCP, protonophore, tigecycline

## Abstract

**Background:** Carbapenems (CAR), colistin (CST), and tigecycline (TGC) alone or in combination therapy has become the last-resort antibiotics for treating infections caused by multidrug resistant (MDR) bacteria. However, resistance to these reserve antibiotics are increasingly being reported worldwide. Hence, the quest to find other agents that will synergistically restore the efficacy of these antibiotics have increased.

**Methods:** Sixty-three clinical *Enterobacteriaceae* isolates comprising of *Klebsiella pneumoniae* (*n* = 24), *Enterobacter* spp. (*n* = 15), *Serratia marcescens* (*n* = 12), *Citrobacter freundii* (*n* = 8), *Escherichia coli* (*n* = 2), and *K. oxytoca/michiganensis* (*n* = 2) with known carbapenem resistance mechanisms and undescribed CST and TGC resistance mechanisms were subjected to broth microdilution and meropenem (MEM) disc synergy test in the presence and absence of carbonyl cyanide m-chlorophenylhydrazine (CCCP), a H^+^ conductor (protonophore).

**Results and conclusions:** Susceptibility to MEM, imipenem (IMP), CST, and TGC was found in only 2, 0, 17, and 9 isolates respectively. Addition of CCCP reversed resistance to CST, TGC, IMP, and MEM in 44, 3, 0, and 0 isolates respectively; CST had the highest mean minimum inhibitory concentration (MIC) fold change (193.12; *p* < 0.0001) post CCCP compared to that of MEM (1.70), IMP (1.49) and TGC (1.16). Eight isolates tested positive for the MEM-CCCP disc synergy test. We concluded that CCCP reverse CST resistance in CST-resistant *Enterobacteriaceae*. Although CCCP is an experimental agent with no therapeutic value clinically, further studies are necessary to decipher the mechanisms underlying the CST-CCCP synergy to inform the development of adjuvants that could be therapeutically effective in CST-resistant infections.

## Introduction

Bacterial resistance to last-resort antibiotics viz., carbapenems (CAR), colistin (CST), and tigecycline (TGC), continues to pose a major clinical challenge to infection treatment and management throughout the world (Osei Sekyere, 2016; Osei Sekyere et al., 2016b). Among Gram-negative bacteria, *Enterobacteriaceae* are commonly implicated in resistance to carbapenems through mechanisms such as carbapenemases, porin downregulation, and/or efflux upregulation (Patel and Bonomo, 2013; Sekyere et al., 2015; Osei Sekyere et al., 2016a). On the other hand, resistance to CST is mediated by lipid A modifications through chromosomal mutations (in *mgrB, pmrAB, phoPQ*, and/or *pmrHFIJFKLM*) or the plasmid-borne *mcr-1/2* gene (Olaitan et al., 2014; Osei Sekyere, 2016; Pragasam et al., 2017). TGC is a glycylcycline that is known to affect protein synthesis by binding to the ribosomal RNA; however, the major mechanism of resistance to TGC is hyperexpression of RND-type efflux pumps (Osei Sekyere et al., 2016b). Mutations in regulatory genes (*soxS, ramA, marA*, and *rarA*) leads to up-regulation of AcrAB-TolC intrinsic RND-type multidrug efflux pumps and TGC resistance (Osei Sekyere et al., 2016b). In addition, acquisition and hyper expression of *oqxAB* exogenous RND-type multidrug efflux genes confers resistance to TGC (Osei Sekyere et al., 2016b; Pournaras et al., 2016).

Due to the importance of the cell envelope in mediating resistance to antibiotics through its physicochemical properties, porin channels, and efflux pumps, attention is being drawn to protonophores such as carbonyl cyanide m-chlorophenylhydrazine (CCCP), which is used as an experimental agent with no therapeutic value clinically (Li et al., 2015), as models to study the interactions between antibiotics and the cell envelope's components (plasma membrane, cell wall and capsule; Spindler et al., 2011; Mohamed et al., 2016; Ni et al., 2016). Protonophores (e.g., CCCP) reduce ATP production and increase membrane permeability in bacteria (Spindler et al., 2011; Park and Ko, 2015; Mohamed et al., 2016; Ni et al., 2016) by interfering with the transmembrane electrochemical gradient and proton motive force (Spindler et al., 2011; Yu et al., 2015).

By depolarizing the plasma membrane and reducing ATP production, protonophores such as CCCP can indirectly affect the activity of proton pumps and cellular metabolism to cause cell death (Spindler et al., 2011; Yu et al., 2015). CAR as β-lactam antibiotics, act on the cell wall by inhibiting peptidoglycan synthesis to cause cell lysis (Sekyere et al., 2015; Mohamed et al., 2016). CST is a polymyxin that acts on lipopolysaccharides of the cell membrane to displace Ca^2+^ ions and lyse the cell membrane (Yu et al., 2015; Mohamed et al., 2016; Osei Sekyere et al., 2016b). Due to the association of the bacterial cell envelope (cell wall, cell membrane, and capsule) with the resistance of these important reserve antibiotics, and the ability of protonophores to influence the permeability and energy of the cell envelope, we sought to investigate the effect of CCCP on the resistance of these antibiotics.

CCCP has been reported to reduce efflux activity in CAR-resistant Gram-negative bacteria such that resistance to CAR was reduced or reversed (Huang et al., 2008). It has also been shown to effectively reduce or reverse CST resistance in some Gram-negative bacteria (Park and Ko, 2015; Ni et al., 2016), and its effect on TGC resistance has been shown to be relatively lower or insignificant (He et al., 2015). For instance, the study by Ni et al. (2016) involved only two *Klebsiella pneumoniae* species out of all the Gram-negative samples/isolates used in that study. Hence, these two *K. pneumoniae* species, of which only one was colistin-resistant, were the only *Enterobacteriaceae* species used. The rest were all Gram-negative non-fermenters such as *Acinetobacter baumannii, Pseudomonas aeruginosa*, and *Stenotrophomonas maltophila*. The study by Park and Ko (2015) also involved only *A. baumannii* and no *Enterobacteriaceae*. Therefore, to our knowledge, this is surely the only study involving a true representation and substantial population of *Enterobacteriaceae*.

Moreover, previous studies did not consider the effect of CCCP on CAR, CST, and TGC simultaneously or in tandem (Park and Ko, 2015; Mohamed et al., 2016; Ni et al., 2016). This study attempts to analyse the interaction between CCCP and CAR, CST, and TGC in reversing IMP, MEM, CST, and TGC resistance from a CCCP-antibiotic synergy perspective with the hope that future studies will investigate potential ionophores that can serve as adjuvants to restore the potency of these reserve antibiotics.

## Materials and methods

### Ethical approval

The Biomedical Research Ethics Committee of the University of KwaZulu-Natal Ethical approved this study under the reference number BE040/14.

### Bacterial strains

A collection of 63 clinical isolates of multiple lineages comprising of *K. pneumoniae* (*n* = 24), *Enterobacter species* (*n* = 15), *Serratia marcescens* (*n* = 12), *Citrobacter freundii* (*n* = 8), *Klebsiella oxytoca* (*n* = 2), and *Escherichia coli* (*n* = 2) with well-described carbapenem resistance mechanisms (mainly *bla*_NDM−1_ and *bla*_GES−5_) and unknown CST and TGC resistance mechanisms were used (Table 1); known and well-described CST (mcr-1/2, mutations and insertional inactivation of *mgrB, pmrAB, phoPQ*, and *pmrHFIJFKLM*) and TGC (mutations in *acrAB, rarA, ramAR, marABR*, and *soxS*) resistance mechanisms were not found in 48 isolates when their genomes were compared to that of wild type strains (TGC-susceptible strains of each specie): *K. pneumoniae* MGH78578 (accession number CP000647); *E. cloacae* NCTC 9394 (accession number FP929040.1); *E. coli* ATCC 25922 (accession number CP009072); *S. marcescens* ATCC 13880 (accession number KN050642.1). The isolates were obtained from a private pathology laboratory in Durban, South Africa. The isolates were sourced from 10 private hospitals in Durban by the private pathology laboratory.

**Table 1:** **Effect of CCCP on the MICs of MEM, IMP, CST, and TGC as well as on the zone diameter of MEM on clinical Enterobacteriaceae isolates**.

**Isolate**	BMD^*^MIC (mg/L)								**WGS^†^/PCR/CNP^‡^-confirmed carbapenemases**	**CCCP-MEM disc synergy test^§^**
	**MEM**	MEM+CCCP (Δ^**^)	IMP^***^	**IMP+CCCP (Δ)**	**TGC^#^**	**TGC+CCCP (Δ)**	**CST^§§^**	**CST+CCCP (Δ)**		
*Escherichia coli* ATCC 25922	≤0.25	≤0.25 (1)	0.25	≤0.25 (1)	≤0.25	≤0.25 (1)	0.5	0.5 (1)	Not determined	−^***^
*K. pneumoniae* ATCC BAA 1706	0.5	≤0.25 (≥2)	0.5	≤0.25 (≥2)	≤0.25	≤0.25 (1)	≤0.25	≤0.25 (1)	Not determined	−
***K. pneumoniae***										
C(UNN_S3)	256	128 (2)	128	64 (2)	4	4 (1)	128	1 (128)	*bla*_NDM−1_	−
D(UNN_S4)	512	128 (4)	128	64 (2)	8	4 (2)	256	0.5 (512)	*bla*_NDM−1_	−
I(UNN_S9)	512	512 (1)	256	256 (1)	2	2 (1)	256	1 (256)	*bla*_NDM−1_	−
J(UNN_S10)	128	128 (1)	256	256 (1)	4	4 (1)	0.5	0.5 (1)	*bla*_NDM−1_	−
3_S2	128	128 (1)	128	128 (1)	2	2 (1)	64	0.25 (256)	*bla*_OXA−232_	−
12_S5	64	64 (1)	128	128 (1)	4	4 (1)	64	0.25 (256)	*bla*_NDM−1_	−
13_S6	128	128 (1)	64	64 (1)	1	1 (1)	≤0.5	≤0.25 (≥2)	*bla*_NDM−1_	−
15_S8	16	16 (1)	128	128 (1)	2	2 (1)	128	1 (128)	*bla*_GES−5_	−
18_S10	512	256 (2)	512	256 (2)	4	4 (1)	256	1 (256)	*bla*_GES−5_	+^†††^
20_S11	256	128 (2)	128	64 (2)	8	8 (1)	64	0.25 (256)	*bla*_NDM−1_	−
21_S12	512	256 (2)	128	128 (1)	4	4 (1)	64	0.5 (128)	*bla*_NDM−1_	−
29_S13	512	256 (2)	128	128 (1)	8	8 (1)	256	0.5 (512)	*bla*_NDM−1_	−
30_S14	256	256 (1)	128	128 (1)	2	2 (1)	128	0.5 (256)	*bla*_GES−5_	−
32_S15	128	128 (1)	32	32 (1)	4	4 (1)	0.5	0.25 (2)	*bla*_NDM−1_	−
34_S16	512	512 (1)	512	512 (1)	2	1 (2)	256	1 (256)	*bla*_GES−5_	−
35_S17	512	256 (2)	512	512 (1)	8	8 (1)	256	1 (256)	*bla*_GES−5_	−
36_S18	128	128 (1)	128	128 (1)	2	2 (1)	64	0.25 (256)	*bla*_GES−5_	+
38_S19	16	16 (1)	32	32 (1)	2	2 (1)	32	0.125 (256)	*bla*_GES−5_	−
46	512	512 (1)	256	256 (1)	1	1 (1)	16	0.125 (128)	*bla*_NDM−1_	−
52_S26	512	512 (1)	256	256 (1)	0.5	0.5 (1)	0.5	0.5 (1)	*bla*_GES−5_	−
53_S27	256	128 (2)	128	64 (2)	4	4 (1)	256	1 (256)	*bla*_NDM−1_	−
60	512	256 (2)	128	128 (1)	1	1 (1)	64	1 (64)	*CNP*^−^^‡‡‡^	+
66	512	512 (1)	256	256 (1)	2	2 (1)	1	0.125 (8)	*bla*_NDM−1_	−
70	512	256 (2)	256	128 (2)	4	2 (2)	256	1 (256)	*CNP+*^§§§^	−
***S. marcescens***										
B (UNN38 _S2)	>512	64 (>8)	512	256 (2)	4	4 (1)	256	1 (256)	*bla*_NDM−1_	−
E (UNN41_S5)	128	64 (2)	128	128 (1)	2	2 (1)	256	0.25 (1024)	*bla*_NDM−1_	+
G (UNN43_S7)	16	16 (1)	512	256 (2)	4	2 (2)	256	4 (64)	*bla*_NDM−1_	−
K (UNN47_S11)	128	64 (2)	256	256 (1)	4	4 (1)	128	2 (64)	*bla*_NDM−1_	−
L (UNN_S12)	128	64 (2)	128	128 (1)	4	4 (1)	64	2 (32)	*bla*_NDM−1_	+
7_S3	64	64 (1)	32	32 (1)	2	2 (1)	128	1 (128)	*bla*_NDM−1_	−
45_S21	32	32 (1)	32	8 (4)	4	4 (1)	128	2 (64)	−	−
56_S29	512	512 (1)	256	128 (2)	2	2 (1)	256	1 (256)	*bla*_NDM−1_	−
59_S30	512	256 (2)	256	128 (2)	2	2 (1)	128	2 (64)	*bla*_NDM−1_	−
67_S33	256	128 (2)	512	256 (2)	4	4 (1)	64	2 (32)	*bla*_NDM−1_	−
68_S34	64	64 (1)	32	32 (1)	2	2 (1)	128	1 (128)	*bla*_NDM−1_	−
71_S36	>512	512 (>1)	128	64 (2)	4	4 (1)	128	2 (64)	*bla*_NDM−1_	−
***E. cloacae*** **(UNLESS OTHERWISE STATED IN THE FOOTNOTE)**										
A^****^(UNN37_S1)	64	64 (1)	32	16 (2)	2	1 (2)	4	1 (4)	*bla*_NDM−1_	−
F^††††^ (UNN42_S6)	512	512 (1)	128	128 (1)	4	4 (1)	128	0.25 (512)	*bla*_NDM−1_	−
H^‡‡‡‡^ (UNN44_S8)	>512	512 (>1)	128	64 (2)	2	1 (2)	64	1 (64)	*bla*_NDM−1_	+
1_S1	2	2 (1)	64	64 (1)	2	2 (1)	128	1 (128)	−	−
16_S9^§§§§^	256	256 (1)	128	128 (1)	2	2 (1)	16	0.25 (64)	*bla*_NDM−1_	−
28	128	64 (2)	64	32 (2)	1	1 (1)	1	0.25 (4)	*bla*_NDM−1_	−
41	512	512 (1)	256	256 (1)	1	1 (1)	256	0.5 (512)	*bla*_NDM−1_	−
43_S20^*****^	>512	512 (>1)	64	64 (1)	4	4 (1)	128	1 (128)	*bla*_NDM−1_	−
49_S24^†††††^	512	256 (2)	128	128 (1)	2	2 (1)	256	2 (128)	*bla*_NDM−1_	+
51_S25	>512	512 (>1)	128	64 (2)	0.25	0.25 (1)	128	1 (128)	*bla*_NDM−1_	−
54	512	256 (2)	128	64 (2)	0.5	0.5 (1)	32	0.125 (256)	*bla*_NDM−1_	−
55_S28^‡‡‡‡‡^	512	256 (2)	256	128 (2)	2	2 (1)	128	0.5 (256)	*bla*_NDM−1_	−
63_S31^§§§§§^	512	256 (2)	64	64 (1)	4	4 (1)	128	1 (128)	*bla*_NDM−1_	−
65_S32	512	512 (1)	256	128 (2)	4	4 (1)	64	0.5 (128)	−	−
74	512	128 (4)	256	128 (2)	1	0.5 (2)	128	2 (64)	*bla*_NDM−1_	−
***E. coli***										
10_S4	>512	512 (>1)	512	512 (1)	4	2 (2)	64	0.125 (512)	*bla*_NDM−5_	−
22	512	256 (2)	256	256 (1)	1	0.5 (2)	128	2 (64)	*bla*_NDM−1_	−
***C. freundii***										
4	>512	512 (>1)	512	512 (1)	0.5	0.5 (1)	1	0.125 (8)	*bla*_NDM−1_	−
9	256	256 (1)	128	128 (1)	2	1 (2)	16	0.5 (32)	*bla*_NDM−1_	−
14_S7	64	64 (1)	32	32 (1)	2	2 (1)	1	0.125 (8)	−	−
17	256	128 (2)	256	128 (2)	1	1 (1)	128	0.5 (256)	CNP+	−
26	512	218 (2)	128	32 (4)	0.5	0.5 (1)	256	0.5 (512)	*bla*_NDM−1_	+
27	>512	512 (>1)	128	64 (2)	0.5	0.5 (1)	32	0.125 (256)	*bla*_NDM−1_	−
48_S23	512	256 (2)	128	64 (2)	2	2 (1)	0.25	0.25 (1)	*bla*_NDM−1_	−
72	512	256 (2)	512	128 (2)	1	1 (1)	128	2 (64)	*bla*_NDM−1_	−
***KLEBSIELLA*** **SPECIES**										
2^******^	2	2 (1)	16	8 (2)	1	1 (1)	32	0.125 (256)	CNP+	−
69_S35^††††††^	512	512 (1)	128	128 (1)	2	2 (1)	64	1 (64)	*bla*_NDM−1_	−

* *Microbroth dilution: EUCAST breakpoints (2016) were used for the interpretation*.

† Whole genome sequencing results. Known resistance mechanisms of tigecycline and colistin could not be found in the isolates.

‡ *Carba NP Test: isolates for which PCR or WGS had not been used to confirm their carbapenemase production were confirmed for carbapenemase production using the Carba NP test*.

§ *This was measured with meropenem (MRP) discs and CCCP, an efflux inhibitor. A difference of more than 5mm between the MRP discs and IMP-CCCP discs zone diameters were interpreted as positive for efflux-mediated resistance*.

** *MIC fold change between antibiotic alone and antibiotic+CCCP i.e. ratio of MIC of antibiotic alone to that of antibiotic+CCCP*.

# *Imipenem. EUCAST resistance breakpoint for imipenem and meropenem (MRP) is >8mg/L; susceptibility breakpoint for both antibiotics is ≤ 2mg/L*.

^‡‡^*Tigeycline. EUCAST resistance breakpoint for tigecycline is >2 mg/L; susceptibility breakpoint is ≤ 1mg/L*.

§§ *Colistin. EUCAST resistance breakpoint for colistin is >2mg/L; susceptibility breakpoint is <2mg/L*.

*** *Negative result: the difference in zone diameter between IMP and IMP-CCCP discs is less than 5 mm*.

††† *Positive result: the difference between the IMP and IMP-CCCP discs' zone diameters is ≥ 5 mm*.

‡‡‡ *Carba NP test negative for carbapenemase production*.

§§§ *Carba NP test positive for carbapenemase production*.

**** *Enterobacter asburiae*.

†††† *Enterobacter species*.

‡‡‡‡ *Enterobacter cloacae complex “Hoffmann cluster III”*.

§§§§ *Enterobacter kobei*.

***** *Enterobacter cloacae complex “Hoffmann cluster IV”*.

††††† *Enterobacter asburiae*.

‡‡‡‡‡ *Enterobacter kobei*.

§§§§§ *Enterobacter asburiae*.

****** *Klebsiella oxytoca*.

†††††† *Klebsiella michiganensis*.

Forty-eight of the isolates had been previously characterized using whole genome sequencing (WGS; bioproject number PRJNA287968) to characterize their resistome, and 12 had been characterized by PCR (Osei Sekyere et al., 2016a) to identify their carbapenem resistance mechanisms; three isolates had been subjected to the Carba NP test (Table 1; Osei Sekyere et al., 2016a,c) to determine if they were carbapenemase producers. CST and TGC resistance mechanisms could not be investigated in 15 isolates that had not been subjected to whole genome sequencing (WGS; Table 1). Five out of the 63 isolates (60, 45_S21, 1_S1, 65_S32, and 14_S7) were non-carbapenemase-producing whilst *K. pneumoniae* ATCC BAA 1706 (KP 1076) and *E. coli* ATCC 25922, which were susceptible to all the antibiotics, were used as controls (Pragasam et al., 2016; Table 1).

### Antibiotics, CCCP, VRP, and RSP

Antibiotics, namely MEM, IMP, CST, TGC, and CCCP, which is used herein as an experimental protonophore; as well as efflux pump inhibitors (EPIs) viz., verapamil (VRP) and reserpine (RSP) in powder form were purchased from Sigma-Aldrich (St Louis, USA). VRP is a calcium (Ca^2+^) channel blocker and RSP is a plant-derived efflux pump inhibitor that block MATE, SMR, and ABC-type efflux pumps, particularly in Gram-positive bacteria and in mycobacteria (by VRP); their use in Gram-negative bacteria is so far minimal although efflux inhibition has been reported (Li et al., 2015; Radchenko et al., 2015; Pule et al., 2016). Solutions of VRP were prepared in deionized water whilst RSP was prepared in dimethyl sulfoxide (DMSO) and CCCP in 50% methanol (v/v) (Pragasam et al., 2016). All solutions were prepared on the day of the experiment and kept protected from the light.

### MICs determinations of CCCP, RSP, VRP, MEM, IMP, CST, and TGC

The broth microdilution method was used for the determination of MICs using the Cation-adjusted Mueller-Hinton broth (Sigma-Aldrich, St Louis, USA). The MICs of CCCP, RSP, and VRP were determined using a randomly selected number (*n* = 27) of isolates and controls (Supplementary Table S1). A sub-MIC of these compounds at final concentrations of 10, 256, and 256 mg/L for CCCP, RSP, and VRP respectively were used in determining their effects on MEM, IMP, CST, and TGC resistance to reduce the possibility of their toxicity being responsible for the change in MEM, IMP, CST, and TGC MIC; the concentration of the inhibitors were constantly kept at the MIC concentrations stated above whilst that of the antibiotics were serially increased (Supplementary Table S1). A sub-MIC of 5 mg/L was used for isolate 29_S13, which had a CCCP MIC of 8 mg/L.

The MICs of the isolates to MEM, IMP, CST, and TGC in the absence and presence of CCCP was determined using a sub-MIC of CCCP (final concentration of 10 mg/L) as already described (Park and Ko, 2015; He et al., 2015). The concentration of antibiotics used per well was increased serially by two-fold whilst that of CCCP was kept constant. EUCAST breakpoints (2016) were used to interpret the results (European Committee on Antimicrobial Susceptibility Testing, 2016).

### MEM-CCCP disc synergy test

Antimicrobial sensitivity testing (AST) using the disc diffusion method with MEM discs (MAST Group, Merseyside, UK) was undertaken as already described for all the 63 isolates (Clinical Laboratory Standards Institute (CLSI), 2009). Two MEM discs were used per plate and 10 μL of 20 mg/L CCCP (Sigma Aldrich, St. Louis, MO, USA) was dispensed on one MEM disc per plate prior to incubation at 37°C for 24 h. A difference of ≥5 mm in zone diameter between MEM discs alone and MEM with CCCP was taken as positive for efflux pump-mediated carbapenem resistance (Table 1; Huang et al., 2008).

### Data and statistical analysis

The MIC fold changes resulting after the addition of CCCP, RSP, and VRP were calculated as the ratio of the MICs of the antibiotic alone to that of the antibiotic plus CCCP. A fold change of ≥8 was adopted as significant. The mean MIC fold change per antibiotic per specie was calculated with the following equation:

$$\begin{array}{l} 1/total\;sample\;size\;(n)\times \;sum\;(MIC\;fold\;change\;\\ \;\times \;frequency\;of\;fold\;change). \end{array}$$

**Figure 1:**
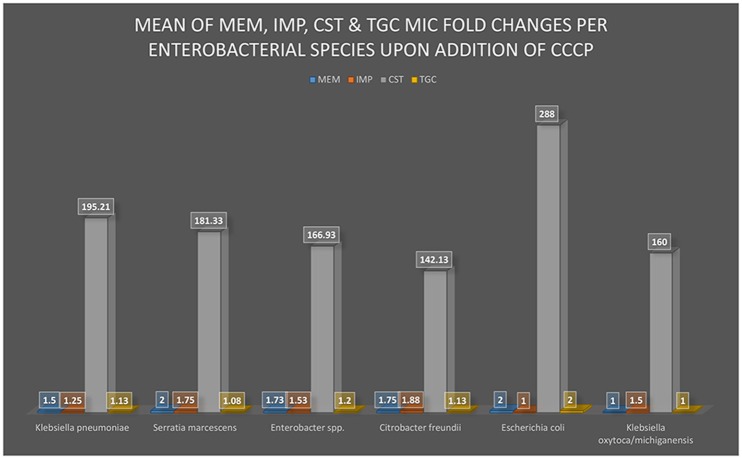
**A graphical view of the mean MIC of MEM, IMP, CST, and TGC fold changes after the addition of CCCP**. The addition of CCCP to the bacterial culture medium either changed or did not change the MIC of MEM, IMP, CST, or TGC to the respective enterobacterial isolates (*K. pneumoniae, S. marcescens, Enterobacter spp*., *Citrobacter freundii, E. coli*, and *K. oxytoca/michiganensis*). The greatest MIC changes occurred in CST with substantial statistical significance (*p*-value was 0.0001> × <0.0099) among all the isolates whilst a statistically insignificant change was observed with all the other antibiotics.

Where the “frequency of fold change” is the number of times a particular MIC fold change was recorded for that antibiotic and specie. Fold changes of >1, ≥2, >8 were used as 1, 2, and 8, respectively, in the mean fold change analysis. The mean fold change per species was translated into a graph using Microsoft Excel™ 2016 (Figure 1).

Results were expressed as mean MIC fold changes of antibiotics per enterobacterial specie upon addition of CCCP (Table 1 and Supplementary Tables S1–S3). The statistical analyses were carried out using non-parametric one-way analysis of variance (ANOVA) followed by Dunnett's multiple comparison test. Fold changes with a *P* < 0.05 were considered statistically significant: ^*^P < 0.05, ^**^P < 0.01 and ^***^P < 0.001. All analyses were performed with GraphPad Prism 5.0 for Windows (GraphPad Software, San Diego, CA, USA).

## Results

### RSP and VRP have no effect on IMP, MEM, CST and TGC resistance

The MICs of CCCP, RSP, and VRP in the various species ranged from 8 to 64, 512 to >512, and 512 to >512 mg/L, respectively (Supplementary Table S1). The concentration of CCCP used (10 m g/L) was well below its MIC except for one isolate that had an MIC of 8 mg/L for which 5 mg/L CCCP was used; hence, CCCP toxicity on the bacterial cells used in this study is minimal or null. Addition of sub-MICs of RSP and VRP to the antibiotics resulted in no change in the antibiotics' MICs (Supplementary Table S1; fold change = 1). Moreover, the influence of MFS, ABC, and SMR efflux pumps that are not inhibited by RSP and VRP in Gram-negative bacteria on these isolates' MICs cannot be excluded: MATE-type and RND types of efflux pumps are believed to be affected by VRP and RSP respectively (Ribera et al., 2002; Shinabarger et al., 2011; Surendranath et al., 2014; Li et al., 2015; Radchenko et al., 2015).

Per the MICs of CCCP, RSP, and VRP alone as well as that of RSP and VRP plus IMP, MEM, CST, and TGC shown in Supplementary Table S1, there were no changes in the MIC of the antibiotics upon addition of the inhibitors. The fold change was one for all species and antibiotics. Due to the mechanism of resistance RSP and VRP as actual EPIs, the role of efflux pumps in conferring resistance to the antibiotics and the probability of CCCP affecting the antibiotics' MICS indirectly through efflux pumps was ascertained.

### CCCP interacts with CST, but not with CAR and TGC

CCCP reversed resistance to CST in 44 isolates and reduced CST MIC by several (from 2 to 1024) folds in almost all the isolates, with a mean fold change of 193.12; 49 isolates had a CST MIC fold change between 64 and 512 and three had no change at all. CST *p*-values ranged from < 0.0099 to <0.0001 in the various species; see Supplementary Table S3. CCCP reversed resistance to TGC in only three isolates, and the overall mean fold change in TGC MICs was 1.16. CCCP could not reverse resistance to MEM and IMP, and their average MIC fold changes were 1.70 and 1.49, respectively. The mean MIC fold change per species is shown in Figure 1 and the *p*-values for CST mean fold change per species are shown in Supplementary Table S3; that of IMP, MEM and TGC are not shown as they were far below the ≥eight-fold change cut off and also had insignificant *p*-values. Susceptibility to TGC, CST, MEM, and IMP in the absence of CCCP was respectively observed in 17, nine, two and zero isolates.

Table 1 summarizes the MICs of MEM, IMP, CST, and TGC alone as well as with CCCP, the carbapenem resistance mechanism of the isolates and the results of the MEM-CCCP disc synergy test. The high level of resistance to MEM (with MICs between 2 and 512 mg/L), IMP (with MICs between 16 and 512 mg/L) and CST (with MICs between 0.5 and 512 mg/L) for all isolates is easily seen from Table 1.

### Four out of five MEM-CCCP positive isolates had a two-fold MIC reduction in MEM

Only eight isolates (E, H, L, 18_S10, 26, 36_S18, 49_S24, and 60) tested positive for the CCCP-MEM disc synergy test (Table 1) and all but two (36_S18 and H) of these had a two-fold reduction in MEM MIC upon addition of CCCP. Isolate 60 was the only CAR-resistant isolate that both produced no carbapenemase and tested positive for the MEM-CCCP test.

### CCCP had no effect on the MEM and IMP MICs of non-carbapenemase-producing isolates

Four of the five CAR-resistant but non-carbapenemase positive isolates (1_S1, 14_S7, 45_S21, 60, and 65_S32) had no change in IMP and MEM MICs; only isolate 60 had an MIC fold change of 2 upon CCCP addition.

## Discussion

Increasing resistance to antibiotics of last-resort is making the research for novel antibiotics as well as for adjuvants that will potentiate the effect of existing ones a present necessity (Osei Sekyere, 2016). Important antibiotics such as β-lactams (e.g., CAR) and CST are known to respectively act on the bacterial cell wall and cell membrane to cause cell lysis and death (Sekyere et al., 2015; Mohamed et al., 2016; Osei Sekyere et al., 2016b). Moreover, RND-type multidrug efflux pump AcrAB-TolC, which is a major contributor to intrinsic multidrug resistance in *Enterobacteriaceae*, is driven by the proton motive force and is found in the cell membrane (Ricci et al., 2014; Li et al., 2015; He et al., 2015). These suggest the important role of the cell wall and cell membrane (with embedded efflux pumps) as potential drug targets and as a bacterial defense mechanism against antibiotics. Due to the activity of CCCP as a protonophore that reversibly binds protons (H^+^) and transports them across the cell membrane, leading to membrane depolarization, eradication of the electrochemical concentration gradient (ECG) and reduced ATP production by ATP synthase (Spindler et al., 2011; Yu et al., 2015; Ni et al., 2016), it was used as an experimental model to investigate how protonophores' effect on the cell membrane could affect the potency of the most important reserve antibiotics: CAR, CST, and TGC.

This study has shown a potential synergy between CCCP and CST in reversing CST resistance among multidrug resistant (MDR) *Enterobacteriaceae*, which can be explored further to find adjuvants that can restore the efficacy of CST in treating CST-resistant infections. Mohamed et al. (2016) recently showed that CST disrupted both the cell membrane and cell wall to cause cell lysis and death, and that the effect of CST on the cell wall is similar to that of β-lactams. Ni et al. (2016) argued that CCCP's depolarization of the cell membrane might restore the negative charges, which are neutralized or reduced in CST-resistant isolates, thus making the resistant cells susceptible to CST again. On the other hand, Park and Ko (2015) proposed that the reduction in ATP production, and subsequently a reduced metabolic activity after treating cells with CCCP, could be responsible for the enhanced activity of CST as no change was observed in the expression levels of *adeABC* and *adeIJK* efflux genes in both resistant and susceptible isolates.

Using the natural producer of CST, *Paenibacillus polymyxa*, Yu et al. (2015) showed that the addition of Ca^2+^, and to a lesser extend Mg^2+^, reduced the bactericidal effect of CST. They thus showed that Ca^2+^ and/or Mg^2+^ depletion was important in enhancing CST activity. Putting these findings together with our own, we hypothesize that CCCP depolarization of the membrane potential and reduction of ATP levels leads to a depletion of or imbalance in Ca^2+^ and/or Mg^2+^ levels in CST-resistant isolates, which facilitates the easier binding of CST to the lipid A to cause cell lysis and death. However, this hypothesis would need to be verified experimentally. The limited role of efflux pumps in conferring CST resistance has also been observed by other researchers (Park and Ko, 2015; Ni et al., 2016). This is not surprising given that CST mainly acts on the lipid A of the outer lipopolysaccharide membrane (Sekyere et al., 2015).

RSP and VRP could not reverse or reduce IMP, MEM, and TGC MICs (Table 1), providing evidence that they are most likely not involved in conferring resistance to IMP, MEM, CST, and TGC (Osei Sekyere et al., 2016b). Notably, the inability of CCCP as well as phenylalanine-arginine β-naphthylamide (PaβN), RSP, and VRP to reverse TGC have been reported, corroborating our findings (Park and Ko, 2015). Thus, the two-fold reduction in TGC MIC by CCCP in 10 isolates as well as the reversal of TGC resistance in three isolates (Table 1) would need further studies to ascertain the mechanism behind this observation.

The possibility that carbapenemases might overshadow the effect of increased efflux pumps activity in the isolates is most likely minimal as RSP and VRP had no effect on the isolates' CAR resistance. Moreover, among the non-carbapenemase producing but CAR-resistant isolates, there was no change in CAR MIC after adding CCCP except in only isolate 60. We hypothesize that the depolarization of the plasma membrane, the subsequent cytoplasmic ion imbalance and reduction of ATP production by CCCP might affect the optimal activity of carbapenemases, which require energy and zinc (in the case of NDM-1) to function (Osei Sekyere et al., 2015) further studies will be necessary to substantiate this.

CCCP enlarged the inhibition zones around the MEM disc on the CCCP-MEM disc synergy test to various sizes (data not shown), albeit a cut-off of ≥5 mm was adopted as positive according to literature (Huang et al., 2008). A pattern of two-fold MIC reduction in MEM after adding CCCP was observed in all but two isolates that tested positive for the MEM-CCCP. Thus, experimental CCCP does have some effect on carbapenem (MEM) resistance that could be investigated further.

Although the peptidomimetic efflux-pump inhibitor, PaβN, is commonly used in Gram-negative bacteria, its effect on CST in *Enterobacteriaceae* has been found to be insignificant (Opperman and Nguyen, 2015; Park and Ko, 2015).

We thus concluded that CCCP reverses CST resistance in CST-resistant *Enterobacteriaceae*. Although CCCP is an experimental agent with no therapeutic value clinically, further studies are necessary to decipher the mechanisms underlying the CST-CCCP synergy to inform the development of adjuvants that could be therapeutically effective in CST-resistant infections.

## Author contributions

JO designed the study, undertook the laboratory work, results analysis and manuscript preparation and formatting. DA took part in the study design and laboratory work, tabulated the BMD results and took part in the revision of the manuscript for scientific merit.

### Conflict of interest statement

The authors declare that the research was conducted in the absence of any commercial or financial relationships that could be construed as a potential conflict of interest.
