# Supplementary material for: Carbonyl Cyanide m-Chlorophenylhydrazine (CCCP) Reverses Resistance to Colistin, but Not to Carbapenems and Tigecycline in Multidrug-Resistant Enterobacteriaceae
Source: Front Microbiol. 2017 Feb 14;8:228. doi: 10.3389/fmicb.2017.00228 (PMC5306282; doi:10.3389/fmicb.2017.00228)
Supplement: Supplementary file 1 [file Table1.DOCX]

**Supplementary Table S1: MICs of CCCP, reserpine (RSP) and verapamil (VRP) alone as well as of RSP or VRP with MEM, IMP, CST and TGC on selected Enterobacteriaceae isolates**

| Isolate | Minimum Inhibitory Concentration (MIC), mg/L | | | | | | | | | | |
| --- | --- | --- | --- | --- | --- | --- | --- | --- | --- | --- | --- |
|  | CCCP | RSP | RSP+MEM (fold change) | RSP+IMP (fold change) | RSP+CST (fold change) | RSP+TGC (fold change) | VRP | VRP+MEM (fold change) | VRP+IMP (fold change) | VRP+CST (fold change) | VRP+TGC (fold change) |
| *Escherichia coli* ATCC 25922 | 8 | >512 | ≤0.25 (1) | ≤0.25 (1) | 0.5 (1) | ≤0.25(1) | >512 | ≤0.25 (1) | ≤0.25 (1) | 0.5 (1) | ≤0.25(1) |
| *K. pneumoniae* ATCC BAA 1706 | 8 | >512 | 0.5 (1) | 0.5 (1) | ≤0.25(1) | ≤0.25(1) | >512 | 0.5 (1) | 0.5 (1) | ≤0.25(1) | ≤0.25(1) |
| ***Klebsiella pneumoniae*** | | | | | | | | | | | |
| C(UNN_S3) | 64 | >512 | >512 (1) | >512 (1) | >512 (1) | >512 (1) | >512 | >512 (1) | >512 (1) | >512 (1) | >512 (1) |
| D(UNN_S4) | 32 | >512 | >512 (1) | >512 (1) | >512 (1) | >512 (1) | >512 | >512 (1) | >512 (1) | >512 (1) | >512 (1) |
| I(UNN_S9) | 16 | >512 | >512 (1) | >512 (1) | >512 (1) | >512 (1) | >512 | >512 (1) | >512 (1) | >512 (1) | >512 (1) |
| J(UNN_S10) | 32 | >512 | >512 (1) | >512 (1) | >512 (1) | >512 (1) | >512 | >512 (1) | >512 (1) | >512 (1) | >512 (1) |
| 15_S8 | 16 | >512 | >512 (1) | >512 (1) | >512 (1) | >512 (1) | >512 | >512 (1) | >512 (1) | >512 (1) | >512 (1) |
| 29_S13 | 8 | >512 | >512 (1) | >512 (1) | >512 (1) | >512 (1) | >512 | >512 (1) | >512 (1) | >512 (1) | >512 (1) |
| 30_S14 | 32 | >512 | >512 (1) | >512 (1) | >512 (1) | >512 (1) | >512 | >512 (1) | >512 (1) | >512 (1) | >512 (1) |
| 32_S15 | 16 | >512 | >512 (1) | >512 (1) | >512 (1) | >512 (1) | >512 | >512 (1) | >512 (1) | >512 (1) | >512 (1) |
| ***Enterobacter cloacae* (unless otherwise stated in the footnote)** | | | | | | | | | | | |
| A^^[[1]](#footnote-1)^^ (UNN37_S1) | 16 | 512 | 512 (1) | 512 (1) | 512 (1) | 512 (1) | 512 | 512 (1) | 512 (1) | 512 (1) | 512 (1) |
| H^^[[2]](#footnote-2)^^ (UNN44_S8) | 64 | 512 | 512 (1) | 512 (1) | 512 (1) | 512 (1) | 512 | 512 (1) | 512 (1) | 512 (1) | 512 (1) |
| F^^[[3]](#footnote-3)^^ (UNN42_S6) | 32 | >512 | >512 (1) | >512 (1) | >512 (1) | >512 (1) | >512 | >512 | >512 | >512 | >512 |
| 1_S1 | 64 | >512 | >512 (1) | >512 (1) | >512 (1) | >512 (1) | >512 | >512 | >512 | >512 | >512 |
| 16_S9^^[[4]](#footnote-4)^^ | 32 | 512 | 512 (1) | 512 (1) | 512 (1) | 512 (1) | 512 | 512 (1) | 512 (1) | 512 (1) | 512 (1) |
| 49_S24^^[[5]](#footnote-5)^^ | 64 | >512 | >512 (1) | >512 (1) | >512 (1) | >512 (1) | >512 | >512 | >512 | >512 | >512 |
| ***Serratia marcescens*** | | | | | | | | | | | |
| B (UNN38 _S2) | 256 | >512 | >512 (1) | >512 (1) | >512 (1) | >512 (1) | >512 | >512 (1) | >512 (1) | >512 (1) | >512 (1) |
| E (UNN41_S5) | 256 | >512 | >512 (1) | >512 (1) | >512 (1) | >512 (1) | >512 | >512 (1) | >512 (1) | >512 (1) | >512 (1) |
| G (UNN43_S7) | 128 | >512 | >512 (1) | >512 (1) | >512 (1) | >512 (1) | >512 | >512 (1) | >512 (1) | >512 (1) | >512 (1) |
| K (UNN47_S11) | 64 | >512 | >512 (1) | >512 (1) | >512 (1) | >512 (1) | >512 | >512 (1) | >512 (1) | >512 (1) | >512 (1) |
| L (UNN_S12) | 64 | >512 | >512 (1) | >512 (1) | >512 (1) | >512 (1) | >512 | >512 (1) | >512 (1) | >512 (1) | >512 (1) |
| 45_S21 | 128 | >512 | >512 (1) | >512 (1) | >512 (1) | >512 (1) | >512 | >512 (1) | >512 (1) | >512 (1) | >512 (1) |
| 59_S30 | 16 | >512 | >512 (1) | >512 (1) | >512 (1) | >512 (1) | >512 | >512 (1) | >512 (1) | >512 (1) | >512 (1) |
| 71_S36 | 128 | >512 | >512 (1) | >512 (1) | >512 (1) | >512 (1) | >512 | >512 (1) | >512 (1) | >512 (1) | >512 (1) |
| ***Citrobacter freundii*** | | | | | | | | | | | |
| 48_S23 | 256 | >512 | >512 (1) | >512 (1) | >512 (1) | >512 (1) | >512 | >512 (1) | >512 (1) | >512 (1) | >512 (1) |
| ***Escherichia coli*** | | | | | | | | | | | |
| 10_S4 | 128 | >512 | >512 (1) | >512 (1) | >512 (1) | >512 (1) | >512 | >512 (1) | >512 (1) | >512 (1) | >512 (1) |
| ***Klebsiella oxytoca*** | | | | | | | | | | | |
| 69_S35 | 64 | 256 | 256 | 256 | 256 | 256 | 256 | 256 | 256 | 256 | 256 |

**Supplementary Table S2: Meropenem, imipenem, tigecycline, and colistin MIC Fold changes after addition of CCCP**

| **ISOLATE** | **MEAN FOLD CHANGE WITH CCCP** | | | |
| --- | --- | --- | --- | --- |
|  | **MEM** | **IMP** | **TGC** | **CST** |
| ***K. pneumoniae*** |  |  |  |  |
| C(UNN_S3) | 2 | 2 | 1 | 128 |
| D(UNN_S4) | 4 | 2 | 2 | 512 |
| I(UNN_S9) | 1 | 1 | 1 | 256 |
| J(UNN_S10) | 1 | 1 | 1 | 1 |
| 3_S2 | 1 | 1 | 1 | 256 |
| 12_S5 | 1 | 1 | 1 | 256 |
| 13_S6 | 1 | 1 | 1 | 12 |
| 15_S8 | 1 | 1 | 1 | 128 |
| 18_S10 | 2 | 2 | 1 | 256 |
| 20_S11 | 2 | 2 | 1 | 256 |
| 21_S12 | 2 | 1 | 1 | 128 |
| 29_S13 | 2 | 1 | 1 | 512 |
| 30_S14 | 1 | 1 | 1 | 256 |
| 32_S15 | 1 | 1 | 1 | 2 |
| 34_S16 | 1 | 1 | 2 | 256 |
| 35_S17 | 2 | 1 | 1 | 256 |
| 36_S18 | 1 | 1 | 1 | 256 |
| 38_S19 | 1 | 1 | 1 | 256 |
| 46 | 1 | 1 | 1 | 128 |
| 52_S26 | 1 | 1 | 1 | 1 |
| 53_S27 | 2 | 2 | 1 | 256 |
| 60 | 2 | 1 | 1 | 64 |
| 66 | 1 | 1 | 1 | 8 |
| 70 | 2 | 2 | 2 | 256 |
|  |  |  |  |  |
| ***Serratia marcescens*** |  |  |  |  |
| B (UNN38 _S2) | 8 | 2 | 1 | 256 |
| E (UNN41_S5) | 2 | 1 | 1 | 1024 |
| G (UNN43_S7) | 1 | 2 | 2 | 64 |
| K (UNN47_S11) | 2 | 1 | 1 | 64 |
| L (UNN_S12) | 2 | 1 | 1 | 32 |
| 7_S3 | 1 | 1 | 1 | 128 |
| 45_S21 | 1 | 4 | 1 | 64 |
| 56_S29 | 1 | 2 | 1 | 256 |
| 59_S30 | 2 | 2 | 1 | 64 |
| 67_S33 | 2 | 2 | 1 | 32 |
| 68_S34 | 1 | 1 | 1 | 128 |
| 71_S36 | 1 | 2 | 1 | 64 |
|  |  |  |  |  |
| ***Enterobacter species*** |  |  |  |  |
| A (UNN37_S1) | 1 | 2 | 2 | 4 |
| F (UNN42_S6) | 1 | 1 | 1 | 512 |
| H (UNN44_S8) | 1 | 2 | 2 | 64 |
| 1_S1 | 1 | 1 | 1 | 128 |
| 16_S9 | 1 | 1 | 1 | 64 |
| 28 | 2 | 2 | 1 | 4 |
| 41 | 1 | 1 | 1 | 512 |
| 43_S20 | 1 | 1 | 1 | 128 |
| 49_S24 | 2 | 1 | 1 | 128 |
| 51_S25 | 1 | 2 | 1 | 128 |
| 54 | 2 | 2 | 1 | 256 |
| 55_S28 | 2 | 2 | 1 | 256 |
| 63_S31 | 2 | 1 | 1 | 128 |
| 65_S32 | 1 | 2 | 1 | 128 |
| 74 | 4 | 2 | 2 | 64 |
|  |  |  |  |  |
| ***Citrobacter freundii*** |  |  |  |  |
| 4 | 1 | 1 | 1 | 8 |
| 9 | 1 | 1 | 2 | 32 |
| 14_S7 | 1 | 1 | 1 | 8 |
| 17 | 2 | 2 | 1 | 256 |
| 26 | 2 | 4 | 1 | 512 |
| 27 | 1 | 2 | 1 | 256 |
| 48_S23 | 2 | 2 | 1 | 1 |
| 72 | 2 | 2 | 1 | 64 |
|  |  |  |  |  |
| ***Escherichia coli*** |  |  |  |  |
| 10_S4 | 1 | 1 | 2 | 512 |
| 22 | 2 | 1 | 2 | 64 |
|  |  |  |  |  |
| ***Klebsiella species*** |  |  |  |  |
| 2 | 1 | 2 | 1 | 256 |
| 69_S35 | 1 | 1 | 1 | 64 |

**Supplementary Table S3: P-values of the mean colistin MIC fold changes upon addition of CCCP in the Enterobacteriaceae isolates**

| **Organism** | **P value with respect to colistin** |
| --- | --- |
| *Klebsiella pneumoniae* | P< 0.0001 |
| *Serratia marcescens* | P< 0.0015 |
| *Enterobacter spp* | P<0.0001 |
| *Citrobacter freundii* | P<0.0039 |
| *Escherichia coli* | P<0.0099 |
| *Klebsiella species* | P<0.0010 |

P≤0.05 is considered statistically significant

1. *Enterobacter asburiae* [↑](#footnote-ref-1)
2. *Enterobacter cloacae complex “Hoffmann cluster III”* [↑](#footnote-ref-2)
3. *Enterobacter species* [↑](#footnote-ref-3)
4. *Enterobacter kobei* [↑](#footnote-ref-4)
5. *Enterobacter asburiae* [↑](#footnote-ref-5)
